# What Are the Best Materials for Filling Children's Teeth?

**Published:** 1900-02

**Authors:** C. Edmund Kells

**Affiliations:** New Orleans, La.


THE
AMERICAN JOURNAL
—OF—
DENTAL SCIENCE.

VOL. XXXIII. BALTIMORE, FEBRUARY, 1900. NO. 10.

### Selections.

ARTICLE I.

### WHAT ARE THE BEST MATERIALS FOR FILLING CHILDREN'S TEETH?

C. EDMUND KELLS, JR., D. D. S., NEW ORLEANS, LA.

A five minute paper upon such a prolific subject must of necessity be concise; hence to the practical treatment of the temporary teeth at once.

These will be divided, from a clinical standpoint, into class: A, good; B, bad; C, very bad.

*Class A.*—Good teeth, of course, are those to which, profiting by our teachings, the mother has given good care and the little patient brought to us at an early age, say two and a half years. At this period the teeth are in perfect order, clean and pleasing in appearance.

The patient is examined every three or four months, and from time to time a spot of decay may appear in the crowns of the molars, which is filled at once with amalgam, however tiny the cavity may be. At the same time, whenever necessary, all the exposed surfaces are polished with orange-wood and pumice, or the engine and a rubber cup may be used. The approximal surfaces are also kept bright by pumice and floss silk.

The incisors, under this care, remain perfect until shed.

The treatment these teeth receive is mostly preventive, and the time (and money) so spent is well invested.

*Class B.*—These patients are brought to us probably after a night of suffering, and say at five years of age, and bespeak at once the neglect they have endured with compound approximal cavities in the molars, pulps nearly or quite exposed, and perhaps an abscess in progress of formation. The incisors also may be involved. The patient is not a type of good health, but nervous, and afraid of everything in the office, ourselves included.

The first step to be taken is to gain the child's confidence, which can only be accomplished by inflicting no pain at the first visit. Some small crown cavity (usually to be found) is slightly prepared and filled with cement, and the patient is greatly encouraged by the idea of having had a tooth filled without pain. By the time all the other work is completed, this filling? is to be removed, the cavity properly prepared and filled with amalgam.

The large compound approximal cavities are in due time prepared as thoroughly as possible, care being taken not to expose the pulps; they are cauterized one or more times with silver nitrate, filled with amalgam if safe, or, if not, with a chlorid of zinc cement. If all progresses smoothly, at the end of six months or a year they may be refilled with amalgam.

Sometimes, when the teeth are crowded, after cauterization, gutta-percha is packed into two facing compound approximal cavities, and allowed to remain for a month or more, when a filling can be more satisfactorily inserted.

If the pulps are exposed after the use of the silver nitrate, a cream of oxid of zinc and creasote is flowed over the exposed portion previous to the insertion of the filling.

These pulps, if thoroughly exposed, will die, but probably painlessly, and the tooth may remain comfortable for some time.

Any tooth in this condition at the age of five will not survive its period of usefulness, but must be lost sooner. However, if the second molars can be held in place, at almost any cost, until the first permanent molars have come through and are well articulated, this should be done, after which their loss is not so serious.

It is not my practice to fill the root-canals of temporary teeth, consequently the treatment of these teeth is merely palliative; when that fails to give relief, extraction is resorted to.

*Class C.*—Under this we will describe a poor little sufferer of two and a half years of age, with all the teeth "rotted to the gums," as we are told, and which description is in fact more accurate than elegant. True enough, the crowns are gone, but in many the pulps are still alive and exquisitely sensitive. Here nothing can equal the cauterization by silver nitrate, which, repeated at intervals, will allay the sensitiveness, and is all that can be done for the little sufferer.

When a girl who has been under my care since childhood marries, she is impressed with the necessity of caring for her children's teeth. She is taught to brush, not rub with a rag, the infant's teeth from the time of their first appearance; at first with clear water, and later, when the child is old enough not to attempt to swallow everything that is put in its mouth, with prepared chalk. Floss silk should also be passed between them daily, and when the child is old enough the mouth should be thoroughly rinsed with diluted lime water night and morning. This constitutes the care the mother is instructed to give the temporary teeth, besides which they should be sent to me for an examination three times annually.

*The Permanent Set.*—The judicious treatment of the first permanent molars is of paramount importance. In my practice there is a tendency to crowded arches, and very frequently the extraction of four teeth becomes advisable.

If the first permanent molars erupt in a defective condition, which they very frequently do, and break down immediately, they are nursed along until the patient reaches the age of eleven, and then extracted. Removing them at this age leaves the twenty anterior teeth for mastication, and allows of the eruption of the second molars in a perpendicular position and almost in the places of the first molars. Meanwhile the bicuspids have dropped back a little, and the entire arch at the age of fifteen is roomy and in good condition.

If the first molars are removed after the appearance of the second molars, these are sure to tip forward more or less, and the crowded condition of the anterior teeth is not so readily overcome, and together, a bad result is sure to obtain.

For nearly twenty years the judicious extraction of the first permanent molars has been practiced by me, and the satisfactory result so obtained is a daily object lesson among those patients who are still in my care.

When the first molars are good, one set of the bicuspids, either the first or second, are sometimes removed with the best results.

As the teeth of the permanent set are erupted they are watched carefully, at least two visits a year being insisted upon. The smallest pits that penetrate through the enamel are filled at once. If these occur before the teeth are fully erupted, they are filled with oxychlorid of zinc first, and later with amalgam or gold. If the teeth are soft, gold is never used. Amalgam is a very satisfactory material for the fissures of bicuspids of children.

Sometimes these buccal and fissue cavities are quite deep, though the enamel margins are quite hard; in which cases the cavities are lined with either gutta-percha or cement, usually the latter, and gold or amalgam completes the filling.

When all is ready for the filling, the cavity is swabbed out with creasote (Morson's only), and then dried with hot air. This will leave the walls in a glistening condition; and if any spot of decay or softened tissue remains, will at once discolor it, and it is removed.

When very deep cavites are found, the enamel borders are prepared thoroughly, but the decay over the pulp is not entirely removed, for fear of exposing the same. This is coated with a cream of oxid of zinc mixed with creasote, and dried and covered with oxychlorid. Later a part of the cement is cut out, and amalgam or gold packed over the remaining protecting layer.

The oxychlorid of zinc is a treatment of the tooth under which the tooth will improve. The zinc phosphate is a filling material only, porous to a certain extent, and does not benefit the tooth otherwise than serving as a filling material.

The approximal surfaces are watched carefully, and upon the slightest suspicion the teeth are wedged apart and the discoloration removed with fine polishing strips; after which the surfaces are brought to a high polish by the use of lampwick carrying moistened pumice. A carrier for this purpose, devised by my father some thirty years or more ago, I have never seen equaled, so I present one for your inspection.

Approximal cavites in children under fifteen years of age are never filled with gold, but with oxychlorid of zinc. Usually the dam is applied, the fillings inserted, patient seated in another room with a book or magazine for from forty-five to sixty minutes, the meantime being employed on other patients, and so is not wasted. The cement is then carefully finished, and paraffin burnished on with a hot instrument.

It is not unusual to find at the age of twelve or thirteen years cavities on the approximal surfaces of all the lower incisors, and even cuspids. Experience and observation have taught me long since that gold will not save such teeth at all, while the above treatment will. I have during this past month filled such teeth with gold for a miss of nineteen years, for whom they were filled with cement in May, 1888, or ten years ago, the cement fillings having wasted but slightly during all this time, and the teeth in the best of condition.

It is essential that a child be taught how to brush the teeth properly, and for this purpose I keep in my cabinet a tooth-brush and set of teeth mounted on continuous gum, and with which I gave a careful object lesson.

Finally, when all other work is completed, the teeth are thoroughly cleansed and polished, and great stress laid upon the necessity for the patient's keeping them in that condition.

I am told that the teeth of children in this section will not stand the same treatment as those in the North. Of this I know nothing personally, but of the utter failure of gold in children's teeth I do not judge by my own work alone. Scores of my little patients go off to school, and have gold fillings inserted by operators of the best reputations, which, however, soon fail. So my hearers of a distant clime must please bear climatic considerations in mind when criticizing my methods, and also my work when it falls under their view.—*Pacific Medico-Dental Gazette.*

